# Investigation of Volumetric Block Proportion (VBP) Effect on Excavation-Induced Ground Response of Talus-like Rock Mass Based on DEM Simulations

**DOI:** 10.3390/ma15248943

**Published:** 2022-12-14

**Authors:** Shuaifeng Wang, Yinlian Yi, Xiaochang Li, Shaoqiang Zhang, Zixin Zhang

**Affiliations:** 1 Department of Geotechnical Engineering, College of Civil Engineering, Tongji University, Shanghai 200092, China; 2 Key Laboratory of Geotechnical and Underground Engineering, Ministry of Education, Tongji University, Shanghai 200092, China; 3 PowerChina Roadbridge Group Co., Ltd., Beijing 100048, China

**Keywords:** talus-like rock mass, volumetric block proportion (VBP), DEM simulation, ground response, tunnel excavation

## Abstract

Due to the complexity of the talus-like rock mass with different values of volumetric block proportion (VPB), it is thus crucial to explore the VBP effect on the excavation-induced ground responses. We conduct a series of 2D DEM (discrete element method) simulations on a common circular tunnel excavation in the talus-like rock mass with different VBPs (0%, 15%, 50%, 85% and 100%). For each VBP, two support scenarios, i.e., unsupported and supported by a rigid lining, are considered. The micro characteristics of the excavation-induced ground responses, including the contact force, force chain, coordination number and shear-slip contact, and the stress distribution and ground settlement are elaborated in detail. Accordingly, three types of talus-like rock masses are identified as soil-, hybrid- and rock-types, corresponding to VBP = 0–15%, 50%, and 85–100%, respectively. It is found that the lining support is essential for maintaining the ground stability of a tunnel excavation in the soil- and hybrid-type talus-like rock masses while the backbones formed by rock blocks in the rock-type talus-like rock mass can provide a certain support for the surrounding ground. Our findings have important implications for optimizing the construction scheme of tunnel excavation in different types of talus-like rock masses.

## 1. Introduction

During the engineering construction, many kinds of different geological materials may be encountered, such as the fractured or blocky rock mass in mountainous terrain [1], soft soil in urban area [2], and sandy-cobble stratum in some special region [3], which have been fully investigated by various methods. Recently, one special type of mixed geomaterials, i.e., soil-rock mixture [4] or bimrocks (short for block-in-matrix rocks) [5], which exhibits complex geological structures, has attracted the attention of many researchers. Many studies have focused on its basic mechanical properties and engineering stability [6,7,8]. In our practical experience of a highway tunnel [9], we found another special kind of mixed rock mass, which is more complicated and more difficult to handle with than the traditional talus formation (soil-rock mixture or bimrocks). Therefore, we called it the talus-like rock mass [9]. This variety of rock mass contains many rock blocks with various sizes and fine grains or soils with different fractions under different burial depth conditions. The sizes and distributions of rocky blocks and grains are more complex than soil-rock mixture due to the intractable fractures and weak interlayers. Therefore, it is of significant importance to investigate the mechanical characteristics of the talus-like rock mass in both the micro and macro perspectives.

In the past decades, many studies have investigated the mechanical properties of soil-rock mixture or similar mixed geomaterials. Kim and Ha [10] found that the larger maximum particle size could enhance the friction angle during the direct shear test of coarse-grained soils. Afifipour and Moarefvand [11] reported that the uniaxial compression strength, Young’s modulus and failure strain decreased with exponential correlation as the rock block proportion increased in the range of 70–90%. Kalender et al. [12] proposed a generalized conceptual empirical approach to predict the strength of unwelded bimrocks and bimsoils (short for block-in-matrix soils) based on the collection and processing of literature data. Chang and Phantachang [13] revealed that the shear strength of gravelly soils is affected by the packing condition and gravel content. In particular, the drained shear strength is decreased compared with the pure sand when the gravel content is less than 20%. Sonmez et al. [14] investigated the influence of block content on the mechanical parameters of welded bimrock mass. Mahdevari and Moarefvand [15] reported that the deformation modulus of remolded bimrock samples increased with the volumetric block proportion (VBP) increment and fractal dimension reduction. Wang et al. [16] summarized an approximate quadratic polynomial relationship between the uniaxial compression strength, elastic modulus and VBP, and a linear piecewise relationship for the internal friction angle, cohesion and VBP. The effects of rock block content and confining pressure on the dynamic characteristics of soil-rock mixture were further investigated [17]. The increasing rock content in soil-rock mixture changes the internal contact force from “between soil and rock” to “between rocks”, and the skeleton formed in the rocks gradually develops overall stiffness [18]. The fluctuations of the post-peak strength become more obvious and the width of shear band increases as the rock block proportion increases [19]. In addition, the block sizes influence the deformation and failure behavior of an artificial welded bimrock significantly. Larger rock blocks develop more tortuous cracks with mixed tensile-shear mode and low peak strength, while the bimrock with small blocks behaves similarly to the intact rock [20,21]. The rock block form or shape is another important factor that influences the shear behavior of soil-rock mixture [22,23]. The models for imperfectly bonded soil-rock mixtures showed that the lower interface stiffness coefficient would decrease the elastic modulus [24]. Napoli et al. [25] proposed a new classification for geotechnically complex formations based on the composition, degree of internal anisotropy, degree of stratal disruption and mixing, and the VPB, which is a systematic theoretical and conceptual study about the geotechnically complex formations in shallow, although no specific mechanical properties investigation was involved. Almost all the afore-mentioned studies involved the VBP or rock content for that it is an important factor affecting the mechanical properties of soil-rock mixture [26]. Further studies have also reported the influence of freeze-thaw cycles [27,28,29] and microbial mineralization [30] on the mechanical properties of soil-rock mixture. As for the talus-like rock mass, our previous study [31] investigated the influences of VBP on the shearing characteristics using large-scale direct shear tests and numerical simulations.

There are plenty of basic investigations on the mechanical properties of soil-rock mixture promote the understanding of the mixed geomaterials and guide the implementation for practical engineering, e.g., slopes and tunnels. Lu et al. [32] analyzed the slope stability of clay-rock mixture, and showed that the factor of safety was enhanced only in the case of rock content greater than 60%. Napoli et al. [33] employed a stochastic approach to investigate the slope stability of bimrocks with block proportions ranging from 0% to 70%, and suggested that bimrocks should be regarded as heterogeneous materials in practical engineering design. Their further studies indicated that the rock shapes and inclination also affected the stability of bimrock slopes when the block content was larger than 40% [34]. Yang et al. [35] carried out the stability analysis of soil-rock mixture slopes and indicated that the higher rock block content would induce higher factor of safety. This is consistent with the rule revealed by Khorasani et al. [36], which also showed that the uncertainty also increased as the VBP increased. Montoya-Araque et al. [37] analyzed the stability of two-dimensional bimslopes (short for block-in-matrix slopes) stochastically based on the novel tortuous surface method. The results showed that tortuous failure surfaces were developed and the increase of factor of safety was noteworthy when the block content exceeded the range of 20–30%. In addition to the rock content, the block size distribution is another important factor affecting the stability of soil-rock mixture slopes, which has a coupling effect with rock content [38]. Liu and Liu [8] simulated the earthquake-induced soil-rock mixture accumulation bodies, and found that cracks appeared in the back edge of the upper accumulation body, which promoted the following collapse and sliding of other bodies. Under the rainfall conditions, an erosion failure was observed in the soil-rock mixture slope, and the increasing rock content would densify the soil skeletons, decrease the migration of fine particles, and finally weaken the erosion effect [39]. The groundwater level also has influences on the stability of bimslopes, i.e., the normalized safety factor was recommended to reduce by 25% when the bimslopes was fully saturated [40]. Large-deformation failure mechanisms of soil-rock mixture slopes were analyzed by Zhao et al. [41]. They revealed that the rock blocks could significantly influence the failure characteristics, inducing heterogeneous plastic zones and multiple sliding regions. Peng et al. [42] reported that the increasing roundness of rock blocks induced larger deformation of soil-rock mixture slopes, and a higher sorting coefficient decreased the deformation. Since the soil-rock mixture or bimrocks usually distribute in the shallow layers in subsurface, the studies and practices for slope stability are encountered more frequently. However, for the tunnel excavation in bimrocks or more complicated talus-like rock mass in depth, there are only a few studies available at present. Napoli et al. [7] investigated the stability of deep circular tunneling in heterogeneous rock mass with a chaotic block-in-matrix fabric with different VBPs, indicating that a larger VBP would promote the tunnel stability. Du et al. [43] established a multi-scale method to simulate the tunnel excavation in sandy cobble stratum. However, they simulated the rock blocks with regular elliptical [7] or circular blocks [43], none of them considered the real angular rock blocks in nature. Qin et al. [44] analyzed the failure modes of a tunnel in loose granular stratum based on DEM (discrete element method) simulations and put forward corresponding construction countermeasures. However, the involved stratum was composed of circular particles and the effects of VBP on the tunnel and ground stability was not included. In fact, our previous study has elaborated the complexity of the talus-like rock mass and engineering challenges in this kind of stratum [9]. More strives are needed to explore the intrinsic characteristics of talus-like rock mass and the ground response induced by tunnel excavation in this special kind of mixed geomaterial, which motivates the development of this current work.

In this paper, we build a series of numerical models containing circular soil particles and polygonal rock blocks to reproduce the talus-like rock mass with different VBPs. A common circular tunnel is considered to be excavated in the rock mass with two support scenarios, i.e., unsupported excavation and supported excavation by a rigid lining. We analyze the microscopic characteristics (contact force, force chain, coordination number and shear-slip contact), stress states and settlements of the ground before and after excavation to investigate the excavation-induced response of the talus-like rock mass and the dependency on VBP. Furthermore, the influences of the ratio of cover depth to tunnel diameter on the excavation-induced ground response with different VBPs are discussed in terms of the ground settlement and shear-slip contacts. Finally, conclusions are drawn.

## 2. Numerical Model

In this paper, we adopt the Particle Follow Code in two Dimensions (PFC2D) [45] to study the influences of volumetric block proportion (VBP) on the excavation-induced ground responses of the talus-like rock mass, i.e., a special type of mixed geomaterial. In PFC2D, the material is constituted by discrete rigid particles with a certain mass based on the principles of DEM. These particles translate, rotate and interact with each other in a given computational domain, which is usually bounded by rigid walls. The micro parameters of particles and contacts dominate the particles’ behavior, which should be calibrated on the basis of laboratory tests to well reproduce the macro behavior of the simulated material.

### 2.1. Model Configuration

**Figure 1:**
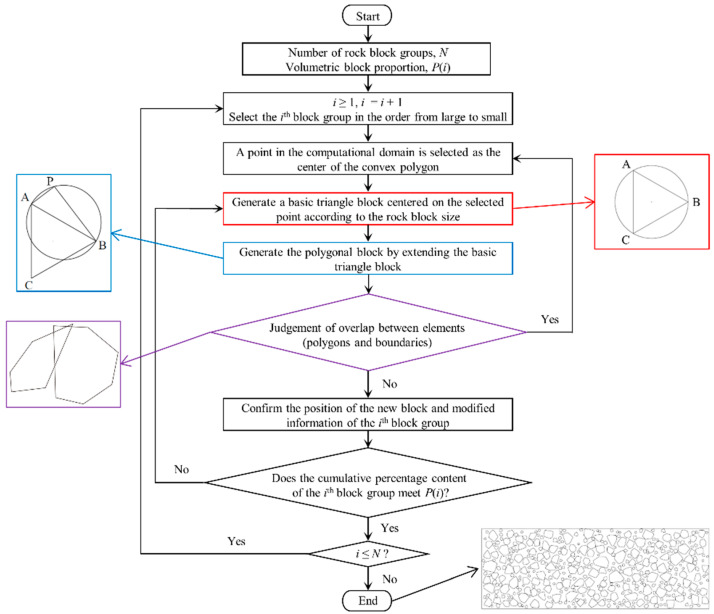
The procedures of generating polygons to model rock blocks.

For this special type of rock-in-soil mixture, the soil and the rock are modeled by particles and rigid blocks (a kind of element in PFC2D), respectively. We use random extension algorithm for polygonal block [46] to generate a series of nonoverlapping convex polygons to model the rock blocks in the talus-like rock mass. The main procedures of the algorithm are shown in Figure 1: (1) generate a basic triangle by randomly locating three non-colinear points in the computational domain, (2) choose an edge of the triangle to generate a circle and select a random point on the circle to generate a new quadrangle, (3) prolongate the quadrangle to generate a predesigned polygon by repeating step 2, (4) check the overlap between the polygon and other elements, e.g., polygons and boundaries, and (5) repeat generating polygons until a predesigned VBP is reached. The polygons are then concerted to rigid blocks in PFC2D, which are filled by disks. Afterwards, disks are generated to fill the voids between blocks and boundaries to model soil particles. The soil particles and rock blocks are assigned different micro parameters to form the final model of the talus-like rock mass, which will be calibrated on the basis of laboratory tests in the following subsection.

### 2.2. Model Calibration

**Figure 2:**
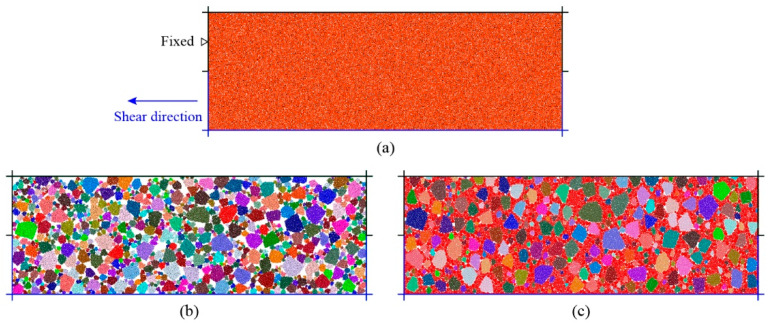
The calibration models for (**a**) soil, (**b**) rocky block and (**c**) mixture samples (after [31]).

The trial-and-error method is usually used to calibrate the micro parameters for DEM simulations due to the unclear relationship between the macro characteristics of materials and the micro behaviors of particles and contacts. In our model, we use the rolling resistance model [22,47] to reproduce the contacts between soil particles and rock blocks. Since the rock blocks may be crushed and broken under loading, the linear parallel bonding model is adopted to simulate this behavior by bonding the disks inside the polygons [22,48,49,50,51]. We conducted a series of large-scale direct shear tests on the samples of talus-like rock mass, which were collected from the Tayi Tunnel of Jian-Ge-Yuan Highway Project in Yunnan province in the Southwestern China [9,31]. Three calibration models, i.e., pure soil, rock blocks and mixture samples, were then established to calibrate the micro parameters (Figure 2). It should be noted that the mixture sample has the VBP same as the natural talus-like rock mass, i.e., 57.9%. The calibrated parameters for both soil particles and rock blocks are listed in Table 1. The simulation results generally agreed well with the experimental tests (see Figure 20 in [31]), indicating that the calibrated parameters can well reproduce the mechanical behaviors of the talus-like rock mass.

### 2.3. Tunnel Excavation Model

Each particle-based DEM model has numerous particles generated in the computational domain. If using the same particle sizes calibrated in Section 2.2 to build a model with a size of dozens of meters containing a circular tunnel of 6 m diameter, there will be dozens of billions of particles, which exceeds the current computing ability. Thus, we adopt the principle of the centrifuge test to reduce the particle number [51,52]. In this model, we generate particles using the parameters listed in Table 1 and set the gravitational acceleration as *N*g, where g is the original gravitational acceleration with a magnitude of 9.8 m/s^2^ and *N* is the similitude ratio (the ratio of a parameter in the numerical model to the one in the prototype model, usually ranges from dozens to hundreds). The size of the numerical model is then set as 1/*N*. As a consequence, the stress state in the numerical model is equivalent to the realistic in-situ stress state.

In order to make a balance between the computational efficiency and the accuracy of the numerical model, we set four assumptions as follows: (1) the initial stress is totally induced by the gravitational field and the tectonic and seepage fields are ignored, (2) the tunnel excavation is simulated by deleting particles inside the excavation space within a single step and the excavation duration is ignored, (3) the tunnel lining is modeled by rigid walls and the convergence deformation of the lining is ignored, and (4) the boundaries are rigid and frictionless.

**Figure 3:**
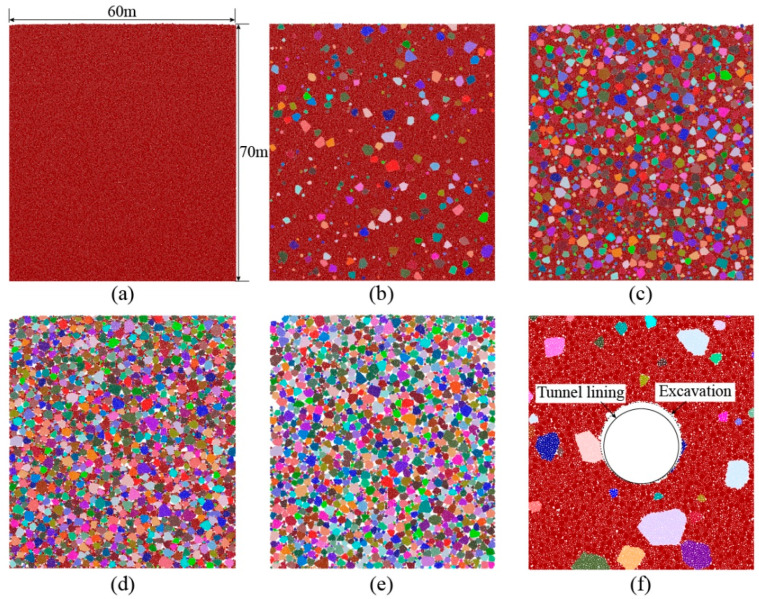
The model setup of the tunnel excavation model: (**a**–**e**) represent VBPs of 0%, 15%, 50%, 85% and 100%, respectively. (**f**) The tunnel excavation model.

As shown in Figure 3, the size of the model is 600 mm × 700 mm, of which two side boundaries and a bottom boundary are launched to restrict the model displacement. The upper surface of the model is not restricted to model the free ground surface. According to the principle of the centrifuge test, we set the similitude ratio as 100, i.e., the height and width represent a computational domain of 60 m × 70 m. We built five models of different VBPs (0%, 15%, 50%, 85% and 100%) as shown in Figure 3a–e. Each model has more than 170,000 particles. A typical circular tunnel with a diameter d = 6.5 m is excavated in the middle of the model by deleting particles inside the excavation space. At the same time, the tunnel lining, i.e., an assembly of rigid walls, is installed. In order to simulate the ground loss due to excavation, the diameter of the excavation is slightly larger than the diameter of the tunnel lining as shown in Figure 3f, where the void spacing is set as 10 cm. Afterwards, the model is executed 0.005 s to investigate the ground responses due to excavation. In order to better capture the excavation-induced responses of the talus-like rock mass, two excavation scenarios, i.e., supported with lining and unsupported, are considered.

### 2.4. Monitoring Scheme and Analysis Parameters

**Figure 4:**
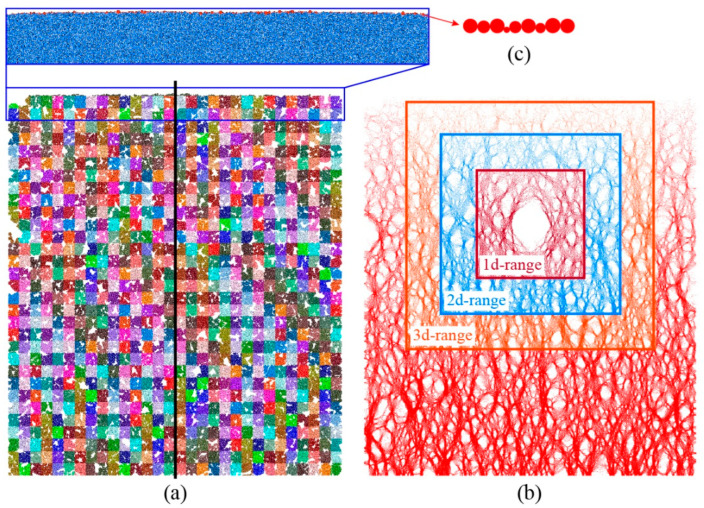
The monitoring scheme of the tunnel excavation model: (**a**) measuring line, (**b**) group division for distribution of contact forces, and (**c**) particles on the surface for measuring ground settlement.

We establish a vertical measuring line in the middle of the model (black line in Figure 4a), along which a series of measuring circles are distributed without overlap. The numerical stress state with respect to the measuring line can be obtained by averaging the stresses inside the measuring circles. The average unit weight $\bar{\gamma }$ of the model is derived as:

(1) $$\bar{\gamma }=\frac{\gamma_{b}\cdot V_{b}+\gamma_{s}\cdot V_{s}}{V_{\mathrm{total}}},$$

where $\gamma_{b}$ and $\gamma_{s}$ are the unit weights of rock block and soil, respectively. In this case, *V*_b_, *V*_s_ and *V*_total_ are the block volume, soil volume and total volume of the materials. The theoretical gravity stress can be derived from the average unit weight times the depth. The model is divided into 30 × 35 squares with a size of 2 m. For each square, we record the average displacement, stress and coordination number. Furthermore, the particles around the tunnel excavation are categorized into three groups to investigate the distribution of contact forces (Figure 4b): 1d-, 2d- and 3d-range groups. We also select a series of particles on the free surface of the model and record their vertical displacements to monitor the ground settlement (Figure 4c).

Based on the stress tensor (*σ_xx_*, *σ_xy_* and *σ_yy_*) recorded in the measuring squares, we derived the maximum and minimum principal stresses as:

(2) $$\sigma_{\max,\min}=\frac{1}{2}\left(\sigma_{xx}+\sigma_{yy}\right)\pm \sqrt{{\left[\frac{1}{2}\left(\sigma_{xx}+\sigma_{yy}\right)\right]}^{2}+\sigma_{xy}^{2}},$$

where *σ*_max_ and *σ*_min_ are maximum and minimum principal stresses, respectively. We further calculate the stress variation angle *α*, i.e., the angle anticlockwise from the positive *x*-direction to the principal stress, as:

(3) $$\alpha =\frac{1}{2}\tan^{-1}\left(\frac{-2\sigma_{xy}}{\sigma_{xx}-\sigma_{yy}}\right).$$

The coordination number is the average number of contacts per particle, representing the packing density of a granular assembly [53,54], which is defined as:

(4) $$C_{n}=\frac{2N_{c}}{N_{p}},$$

where *N*_c_ and *N*_p_ are the numbers of contacts and particles, respectively.

## 3. Results

### 3.1. General Observation before Tunnel Excavation

**Figure 5:**
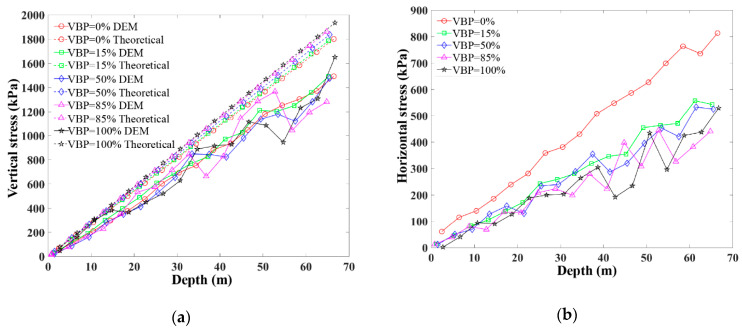
The stress distribution along the measuring line before tunnel excavation in the talus-like rock mass with different VBPs: (**a**) vertical stress and (**b**) horizontal stress.

The theoretical and numerical gravity stresses are shown in Figure 5a, where both of them increase linearly with respect to the depth. Within the same depth, the theoretical gravity stress has a general positive relationship with VBP. For small values of VBP, i.e., 0% and 15%, the numerical gravity stress increases with the depth in a general linear way. However, the gravity stress of the ground with a larger VBP (≥50%) seems to fluctuates more obviously in a larger depth. Moreover, the fluctuation is more significant in a larger VBP case. This phenomenon may be attributed to the heterogeneity and noncontinuity induced by irregular rock blocks. The numerical gravity stress is smaller than the theoretical one in each VBP case, which is induced by the larger void ratio of the numerical model than the realistic one. As shown in Figure 5b, the horizontal stress of the soil formation, i.e., VBP = 0%, is larger than other cases. The fluctuation characteristics of the horizontal stress are similar to the vertical one. The fluctuation is more visible when the depth or the VBP goes larger.

### 3.2. Microscopic Perspective of Ground Responses

#### 3.2.1. Contact Force and Force Chain

**Figure 6:**
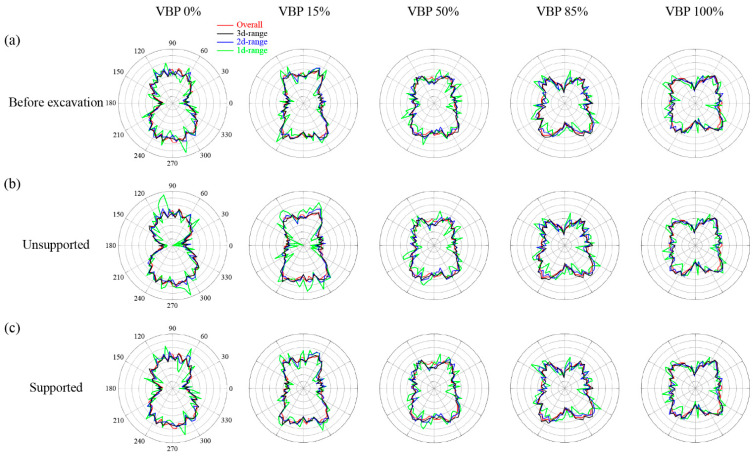
The proportion of the contact force in each direction in different groups: (**a**) before excavation, (**b**) unsupported excavation, and (**c**) supported excavation.

**Figure 7:**
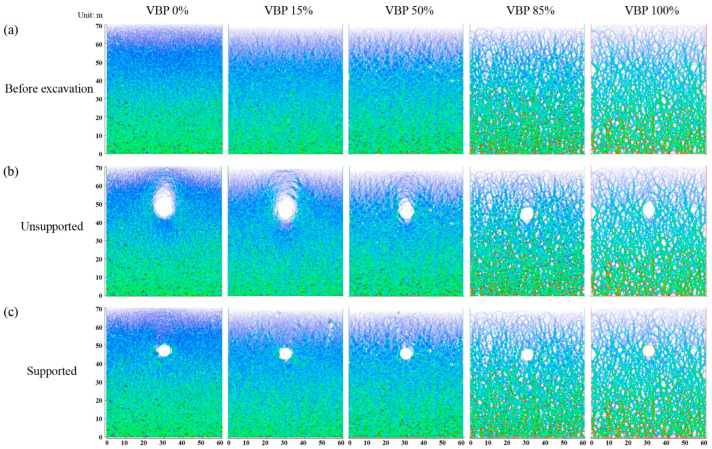
The force chains generated in the (**a**) original ground before excavation, (**b**) excavation without support, and (**c**) supported excavation by lining.

We traverse all the contacts and examine the directions of contact forces to plot the proportion of the contact force in each direction in different groups (Figure 4a) as well as the overall distribution by counting all the contact forces within the computational domain as shown in Figure 6. Before excavation, the distribution of the contact force directions in the 1d-range group vary more violently than the overall trend while the contact forces in the 2d- and 3d-range groups generally have an approximate distribution as the overall trend (Figure 6a). When the VBP is small, i.e., ≤50%, the vertical contact stresses are more prominent than the horizontal ones. As the VBP exceeds 85%, the horizontal contact stresses become more prevailing. The shape of the component diagram varies from a prolonged ellipse to a quasi-circle. Generally, the proportion of horizontal contact stresses increase with the increasing VBP. On the contrary, the proportion of vertical contact stresses has a negative relationship with VBP. This trend can be also observed from Figure 7, where the horizontal force chains become more obvious as VBP increases. The variation in the contact stress directions implies that the talus-like formation behaves more similar to a soil ground when VBP is small while the contact stresses are dominated by the structural characteristics due to ubiquitous irregular rock blocks. Moreover, the force chains become coarser in a larger depth, which is consistent with the stress distribution shown in Figure 5.

After the tunnel excavation (Figure 6b,c), the contact forces in the 3d-range group have a similar distribution compared with the overall pattern in both the unsupported and supported scenarios, showing that the model size is sound to eliminate the boundary effect. In the unsupported tunnel excavated in the talus-like rock mass with a small VBP (≤50%), sparser force chains are generated just above and below the excavation whereas denser chains are formed on the both sides of the tunnel (Figure 7b). The void above the excavation, which is induced by the collapse of soils and blocks, is more obvious in a smaller VBP. In these cases, an apparent arching effect can be observed above the excavation. However, as the VBP exceeds 85%, the force chains around the tunnel do not show a great change compared with the initial state before excavation. Such a phenomenon is attributed to the complex backbones formed by polygonal blocks in the matrix, which contact and interlock with each other, providing a support for the particles and blocks around the excavation. As a consequence, no obvious void is observed in these cases. As for the supported excavation, the stress distributions in Figure 6 and the force chains in Figure 7 exhibit little difference with the initial state, indicating that the rigid lining performs well to support the surrounding formation.

#### 3.2.2. Coordination Number

**Figure 8:**
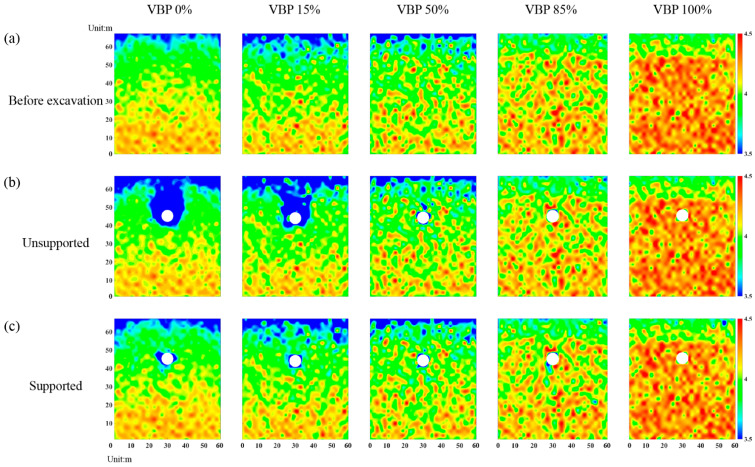
The coordination number distribution in the (**a**) original state, (**b**) excavation without support and (**c**) supported excavation by lining.

**Figure 9:**
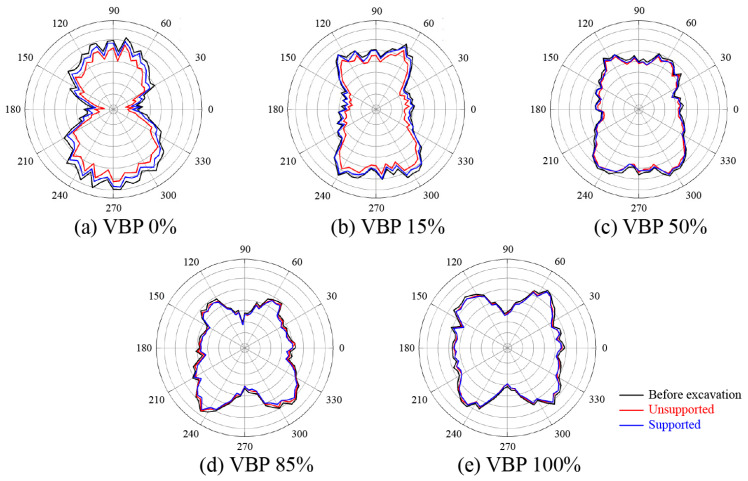
The number of contacts with the same normal vector in each direction within the whole computational domain of VBP = (**a**) 0%, (**b**) 15%, (**c**) 50%, (**d**) 85% and (**e**) 100%.

The coordination number derived by Equation (4) represents the quantity of effective contacts between particles and the relative density of the backbones formed in the talus-like rock mass. In Figure 8, we plot the average value of the coordination number inside each measuring square (Figure 4). Before excavation (Figure 8a), the coordination number increases with the depth and VBP, which agrees well with the phenomenon shown in Figure 7a. In the unsupported scenario, the coordination number below the tunnel keeps its distribution in all the cases with different VBPs (Figure 8b). When the VBP is small, e.g., 0% or 15%, the coordination number around and above the tunnel reduces shapely compared with the initial state, showing that the excavation loosens particles and blocks within this range. This underlines the void in the force chains generated above and around the tunnel (Figure 7b). When the VBP increases, the reduction only arises around the tunnel within a limited range. Moreover, the reduction range becomes smaller as the VBP increases. Around the supported tunnel, the ground is well resisted by the rigid lining and the reduction in coordination number only occurs in a localized region (Figure 8c). By comparing each column of Figure 8, it can be observed that the variation in coordination number in small VBPs (0% and 15%) is more dominated by the support condition. However, when the VBP increases, the variation becomes more inconspicuous no matter whether the tunnel is supported or not. Furthermore, we count the number of contacts with the same normal vector in each direction as shown in Figure 9. For all the cases of different VBPs, the tunnel excavation does not alter the shape of the curve. In the soil formation, i.e., VBP = 0%, the curve shrinks after excavation and its shape in the unsupported case is the smallest (Figure 9a). As the VBP increases to 15%, only the unsupported excavation alters the number of contacts (Figure 9b). For the talus-like rock mass with VPB exceeding 50%, the support condition does not affect the number of contacts. Incorporated with Figure 6b, the proportion of vertical contact forces within the 1d-range group increases, i.e., the proportion of horizontal contact forces decreases, after unsupported excavation in the ground with a VBP of 0% or 15%. As the VBP increases, the directions of contact forces keep the distribution after unsupported excavation. It can be drawn that the force transmission mechanism in the cases of VBP ≤ 15% is more dominated by the support condition whilst the tunnel excavation in the talus-like rock mass with VBP ≥ 50% exhibits limited influence on the force transmission.

#### 3.2.3. Shear-Slip Contact

**Figure 10:**
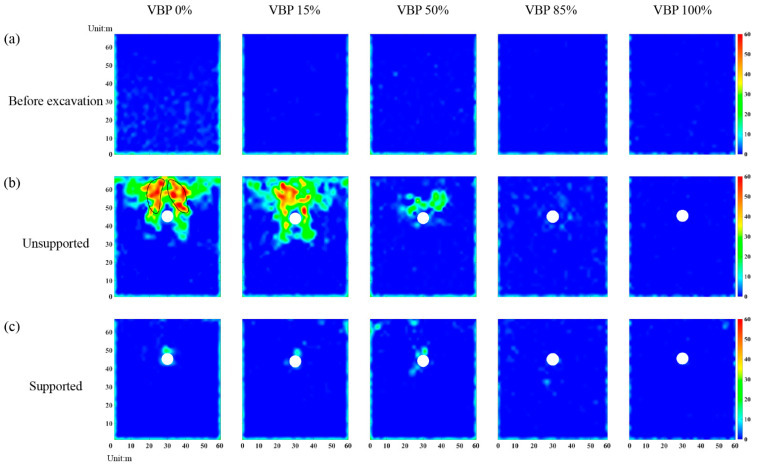
The distribution of shear-slip contacts in the (**a**) original state, (**b**) excavation without support and (**c**) supported excavation by lining. The black curve stands for shear band.

By counting the number of contacts where the shear slip emerges in each measuring square (Figure 4), the area where particles have a large movement after tunnel excavation can be obtained. Accordingly, Figure 10 illustrates the distribution of the number of shear-slip contacts. When the tunnel is excavated without supported in a soil formation (VBP = 0% in Figure 10b), the shear-slip contacts are mainly distributed around the tunnel. On the both shoulders of the tunnel, two obvious shear bands can be identified (black curves in Figure 10b). When the VBP increases to 15%, the shear-slip zone becomes smaller and the shear bands tends to be difficult to recognize. As the VBP increases to 50%, the shear-slip contacts only occur in a narrow range above the tunnel. For VBP = 85% or 100%, no obvious shear-slip contacts are generated around the tunnel. In general, the number of shear-slip contacts has a negative relationship with the VBP. As for the supported tunnel, only a concentrated range above the tunnel has shear-slip contacts when VBP ≤ 50%, while there is basically no apparent shear-slip contact as VBP increases.

From the microscopic perspective of ground responses after unsupported excavation, it can be drawn that: for small VPBs (0% and 15%), the surrounding formation after excavation behaves similarly to a typical soil-type formation [55,56]. For large VBPs (85% and 100%), the structural backbones formed by increasing blocks reduce the microcosmic contacts and provide an effective support for the surrounding rock mass, which has also been revealed by Napoli et al. [7]. When VBP has a moderate value, i.e., 50%, the talus-like rock mass exhibits some hybrid characteristics. For the supported excavation scenario, the ground responses of the talus-like rock mass with VBP ≤ 50% exhibit a few differences from the initial state before excavation, whilst larger VBPs may not cause distinction in the microscopic characteristics after excavation.

### 3.3. Stress Distribution

#### 3.3.1. Horizontal and Vertical Stresses

**Figure 11:**
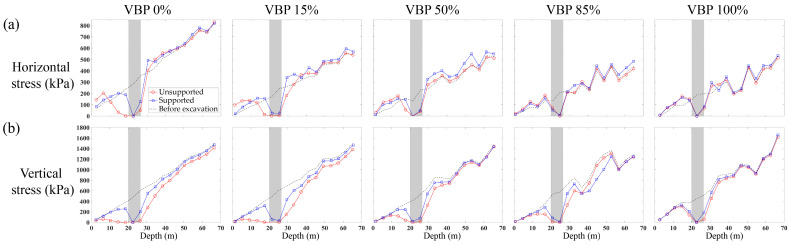
The distribution of (**a**) horizontal and (**b**) vertical stresses along the measuring line. The grey shadow indicates the excavation range.

As shown in Figure 11a, the horizontal stress along the measuring line (Figure 4) after excavation decreases within a certain range above and below the excavation space, while it increases in the area farther away from this range. For the region far away from the tunnel, the horizontal stress after excavation is close to that before excavation. Near the tunnel excavation space, the vertical stress also decreases after excavation (Figure 11b). However, beyond this range, it is still smaller than the initial vertical stress, which is opposite to the horizontal stress. The inconsistency in the horizontal and vertical stresses indicates that the vertical stress above and below the excavation are transferred to the horizontal one, i.e., a formation arching is formed in these ranges, which underlines the observation from the force chains shown in Figure 7. It can be also observed that the arching effect is more obvious in the unsupported scenario with the same VBP and becomes weaker as the VBP increases. Furthermore, the variation range of stresses seems to tend to be smaller as the VBP increases, implying a lessened excavation-induced impact on the stress state of the formation. In addition, the influence of support condition on the stress variation becomes less predominant with an increasing VBP. In particular, in the ground formed by pure rock blocks, i.e., VBP = 100%, there is no obvious distinct between the cases with and without support.

**Figure 12:**
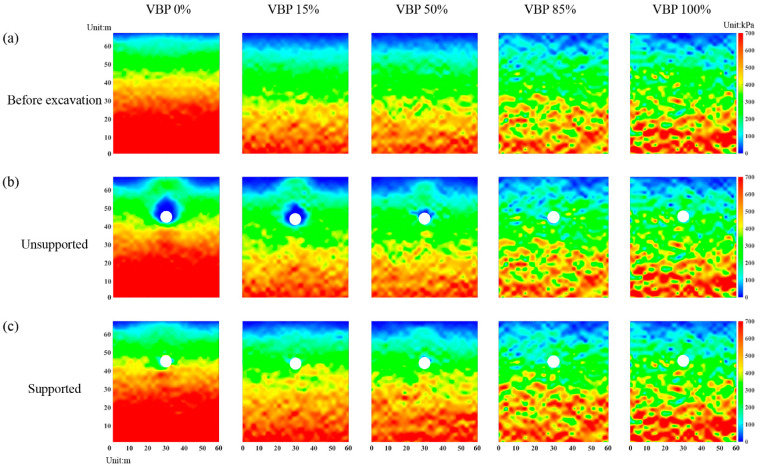
The distribution of horizontal stresses in the computational domain: (**a**) initial state before excavation, (**b**) unsupported tunnel excavation, and (**c**) supported tunnel excavation by rigid lining.

**Figure 13:**
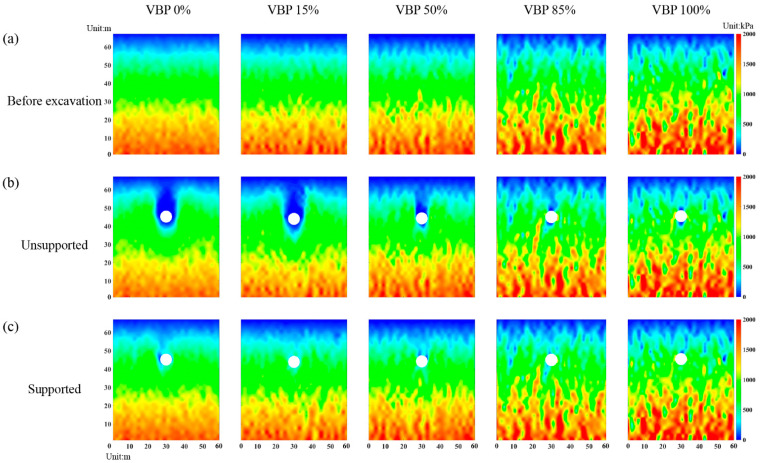
The distribution of vertical stresses in the computational domain: (**a**) initial state before excavation, (**b**) unsupported tunnel excavation, and (**c**) supported tunnel excavation by rigid lining.

We further plot the distributions of horizontal and vertical stresses in the whole computational domain in Figure 12 and Figure 13, respectively. In the unsupported scenario in a soil-type formation (Figure 12b), i.e., VBP = 0 and 15%, the horizontal stress decreases within a certain range formed by the vault and both sides of the tunnel, while the one near the ground surface above the vault increases compared with the initial state (Figure 12a). The reduction range of the vertical stress extends to the surface with a chimney shape (Figure 13b). When VBP = 50%, the reduction ranges of the horizontal and vertical stresses around the excavation both shrink. In particular, the vertical stress on both sides of the tunnel keeps its value after excavation and only the one in the vault and invert decreases. As the VBP reaches 85% or a higher value, the horizontal stress after excavation is same as the initial state before excavation (Figure 12b). As for the vertical horizontal stress, the variation is only concentrated in limited ranges above and below the tunnel, and the reduction region does not reach the free surface (Figure 13b). In addition, the distributions of the horizontal and vertical stresses in the excavated formation of VBP = 0% are approximatively axisymmetric, while those in the talus-like rock mass with larger VBPs exhibit obviously bias and deflection, i.e., they are asymmetric. This may be attributed to the irregular and asymmetric skeletons formed by polygonal rock blocks. Such an asymmetric phenomenon was also captured in the excavation-induced responses of sandy formation constituted by prolonged soil particles [55]. When the distributions of the horizontal and vertical stresses are merged, a range just above the tunnel vault where both the orthogonal stresses decrease can be identified, i.e., a cavity of stress release, which is consistent with the void observed in Figure 7. Above this range, the horizontal stress increases whilst the vertical one decreases, same as the observation shown in Figure 11, i.e., a formation arching is generated within this region. As shown in Figure 12c and Figure 13c, the lining only affects the excavation-induced variation in stress state in the talus-like rock mass with VBP ≤ 15%. As the VBP increases, the impact of lining support becomes feebler. This is similar to the phenomenon captured in Figure 7 and Figure 11.

#### 3.3.2. Rotation of Principal Stress

**Figure 14:**
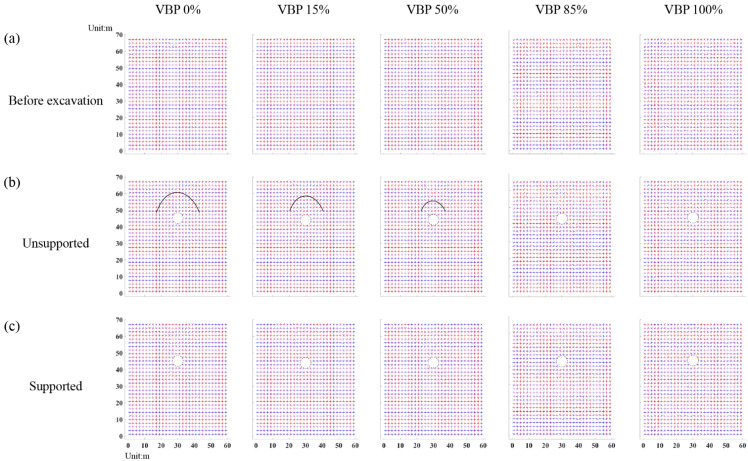
The directions of the principal stresses in the ground of (**a**) initial state before excavation, (**b**) unsupported tunnel excavation and (**c**) supported tunnel excavation. The red and blue segments indicate the maximum and minimum principal stresses, respectively. The black curve indicates the arching formed above the tunnel excavation.

**Figure 15:**
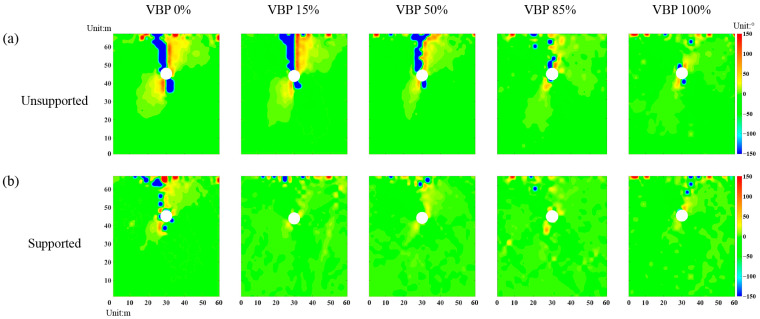
The distribution of the stress variation angle in the ground of (**a**) unsupported tunnel excavation and (**b**) supported tunnel excavation.

By averaging the stress tensors in the measuring squares (Figure 4), the principal stresses are derived by Equation (2) and plotted in Figure 14, where the red and blue segments indicate the maximum and minimum principal stresses, respectively. Before excavation, the maximum principal stress of the strata with different VBPs mainly directs vertically (Figure 14a). In the regions above and below the unsupported tunnel, the principal stresses in the ground with VBPs ≤ 50% rotate to form an apparent arching (black curves in Figure 14b). We further plot the distribution of the stress variation angle derived by Equation (3) in Figure 15, where the maximum principal stress on the upper right and lower left of the tunnel rotates clockwise while the one on the lower right and upper left rotates counter-clockwise. The ranges where the principal stresses rotate clockwise or counter-clockwise extend to the free surface. When VBP = 85% or 100%, the rotation in principal stresses only occurs in very limited ranges above and below the tunnel (Figure 14b and Figure 15a), where the arching effect can be barely observed. In these cases, the skeletons formed by irregular blocks effectively support the surrounding ground and are beneficial to the excavation stability, which is consistent with the observations by Napoli et al. [7]. As for the supported excavation (Figure 14c and Figure 15b), only the principal stresses in the ground of VBP = 0% are well-marked as rotation close around the tunnel while the other cases do not witness apparent stress rotation.

**Figure 16:**
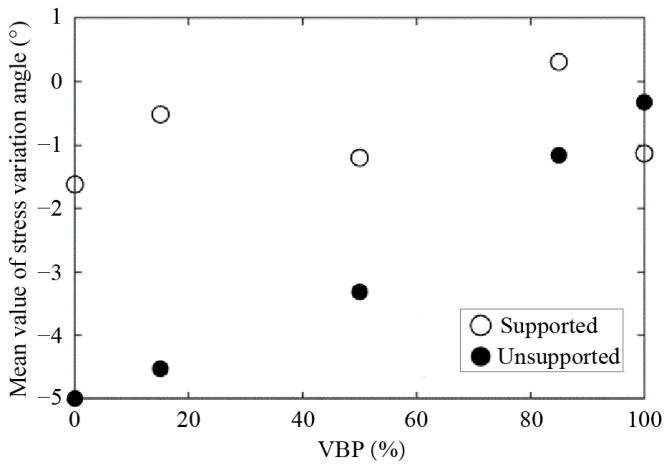
The mean value of stress variation angle distributed in the talus-like rock mass as a function of VBP.

We then calculate the mean value of the stress variation angle within the whole computational domain as shown in Figure 16. In the unsupported scenario, the average stress variation angle has a linearly descending relationship with VBP. As for the supported excavation, the values generally increase with the increasing VBP in a fluctuation pattern. The average stress variation angle in the supported case is smaller than the one in the unsupported case when VBP ≤ 85%, while the relationship reverses in the ground formed by pure blocks. Incorporating this with the observations in Figure 14 and Figure 15, it can be drawn that the rock blocks in the talus-like rock mass with larger VBPs are interlocked by each other to form stable backbones, which will restrict the movement and rotation of particles and blocks, also resulting in less obvious variation in the stress state (Figure 12 and Figure 13) and microscopic contacts (Figure 6, Figure 7, Figure 8 and Figure 10).

### 3.4. Ground Settlement

**Figure 17:**
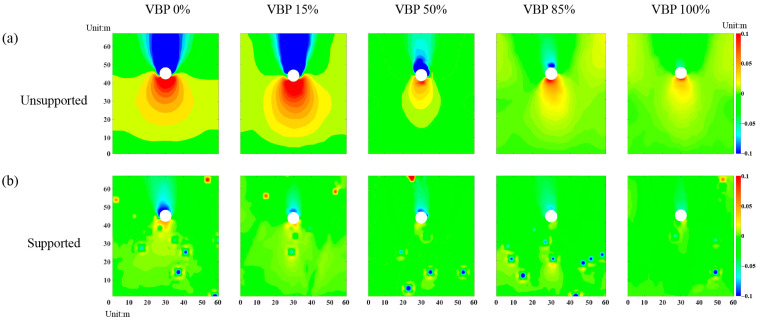
The distribution of vertical displacement in the talus-like rock mass with (**a**) unsupported tunnel excavation and (**b**) supported tunnel excavation.

Figure 17 demonstrates the distribution of vertical displacement in the talus-like rock mass with different support conditions. As shown in Figure 17a, the ground above the unsupported tunnel subsides while the one below the tunnel raises. In addition, the deformation range generally has a negative relationship with VBP. Figure 17b shows that the lining support can effectively limit the excavation-induced ground deformation. The chimney-shaped settlement range (Figure 17a) agrees well with the void of force chains (Figure 7b), reduction ranges of coordination number (Figure 8b) and vertical stress (Figure 13b) as well as the rotation range of the principal stresses (Figure 15b). Additionally, the settlement ranges (Figure 17a,b) are also consistent with the distribution ranges of shear-slip contacts (Figure 10b,c) and the variation ranges of the horizontal stress (Figure 12b,c). These coincidences imply that the excavation-induced ground responses observed in a microscopic perspective have a strong linkage with the macroscopic characteristics.

**Figure 18:**
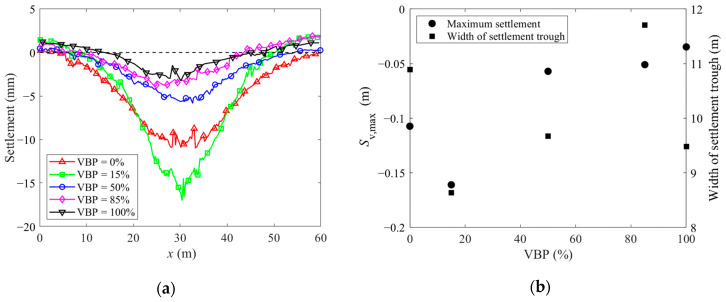
(**a**) The ground settlement curves with different VBPs. (**b**) The maximum settlement and width of the settlement trough as a function of VBP.

We then plot the ground settlement after unsupported excavation in Figure 18a. The Peck formula is further adopted to fit the settlement curve $S_{v}\left(x\right)$ in Figure 18b as follows:

(5) $$S_{v}\left(x\right)=S_{v,\;\max}\cdot \exp\left(-\frac{x^{2}}{2i^{2}}\right),$$

where $S_{v,\;\max}$ is the maximum settlement, *i* is the width of the settlement trough, and *x* is the horizontal coordinate. The case of VBP = 15% has the greatest ground settlement but the narrowest settlement trough. For the soil-type ground of VBP = 0% or 15%, the settlement is enlarged as VBP increases while the excavation-influenced settlement range is contracted at the same time. The settlement trough is similar to the one in granular soil formation [56,57]. For the talus-like rock mass with VBP ≥ 85%, both the ground settlement and the width of the settlement trough exhibit a negative relationship with VBP. The ground with a middle value of VBP = 50% also has the moderate settlement magnitude and range.

## 4. Discussion

**Figure 19:**
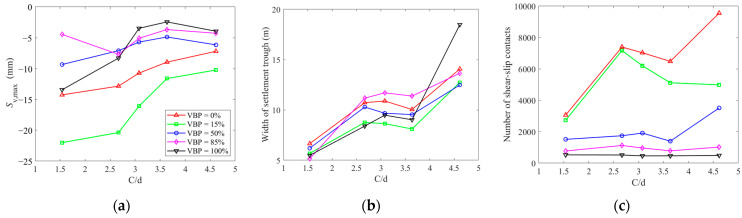
The fitting results for the ground settlements using Peck formula as a function of C/d: (**a**) maximum ground settlement, (**b**) width of settlement trough, and (**c**) number of shear-slip contacts as a function of C/d.

In this paper, we built a series of 2D DEM models consisting of angular rock blocks to reproduce the talus-like rock mass with different VBPs. The excavation-induced ground responses were investigated in the aspects of the microscopic characteristics, stress states and ground settlements. Generally, the talus-like rock mass with VBP ≤ 15% behaves similarly to a typical soil-type formation in terms of the development of force chains, coordination numbers and shear-slip contacts as well as the soil arching. When the VBP reaches 85% or higher, the ground responses in the talus-like rock mass are more predominant by the irregularly-developed backbones by increasing polygonal rock blocks, which can be called rock-type formation. When the block content is in a moderate level, i.e., VBP = 50%, the talus-like rock mass exhibits some hybrid characteristics related to both the soil- and rock-type formations. In addition to VBP, the ratio of cover depth to tunnel diameter (C/d) has also been proven as a significant factor on the excavation-induced ground responses in soil-type formations [57,58,59,60]. Therefore, we further examine the influences of C/d on the ground responses by varying the buried depth and tunnel diameter as listed in Table 2. Figure 19a,b plot the fitting results of the ground settlement using Peck formula derived as Equation (5). Both the maximum ground settlement and the width of the settlement trough with different VBPs generally exhibit a linear relationship as a function of C/d. However, the relationships between the two parameters and VBP are not well-marked as a monotonic law. Figure 19c sums the number of shear-slip contacts generated in the whole computational domain. The number is positively related to C/d only in the soil formation, i.e., VBP = 0%. However, the interrelationship seems unclear for other cases with VBP ≥ 15%. If keeping the value of C/d, the number of shear-slip contacts decreases as VBP increases. Therefore, the content of rock blocks in the talus-like rock mass plays a dominant role on the microscopic characteristics while C/d makes a greater contribution on the macroscopic excavation-induced ground responses. Another observation that can be seen is that the variations in the maximum settlement and the number of shear-slip contacts in the cases of VBP = 0% and 15% are more violent than those in other cases, implying that C/d is of a more significant importance on the soil-type talus-like rock mass, which is consistent with the preceding observation [57,59].

From the diverse characteristics of the excavation-induced responses of the talus-like rock mass in unsupported and supported scenarios, the variation ranges in analysis parameters around the unsupported tunnel are relatively larger than those in the supported case, which is limited in a very narrow area around the excavation. The differences between the supported and unsupported scenarios imply that a rigid lining is effective for maintaining the ground stability of a tunnel excavation. However, the immutable convergence of the rigid support may lead to massive external loads from the surrounding rock mass on the lining, which will then suffer large internal forces. In the practical engineering excavated in rock mass, the New Austrian Tunnelling Method [2,61] recommends that the primary lining should be flexible enough to fully release the in-situ stress around the tunnel in a short-term duration after excavation and the secondary lining is subsequently installed to provide a long-lasting support. Therefore, it is recommended to investigate the ground responses due to tunnel excavation supported by a flexible lining in the future.

## 5. Conclusions

In this paper, we conducted a series of 2D DEM simulations based on the principle of the centrifuge test to explore the effect of VBP on the ground responses due to the excavation of a common circular tunnel in the talus-like rock mass. The random extension algorithm for polygons was adopted to generate nonoverlapping rock blocks and the soil particles were modeled by rigid circular disks. The micro parameters of the blocks and particles, as well as the contacts, were calibrated on the basis of large-scale direct shear tests. We examined five values of VBPs, i.e., 0%, 15%, 50%, 85% and 100%. We demonstrated the microscopic characteristics (contact force, force chain, coordination number and shear-slip contact) of the excavation-induced responses of the talus-like rock mass with varying VBP. The stress distribution and ground settlement were also investigated. Finally, we discussed the influences of the ratio of cover depth to tunnel diameter on the excavation-induced ground responses with different VBPs from both the micro and macro perspectives. The primary conclusions are drawn as follows.

(1) For small values of VBP (0% and 15%), the talus-like rock mass behaves similarly to the typical soil formation in terms of both the micro and macro excavation-induced responses, which can be called soil-type talus-like rock mass. When VBP ≥ 85%, the excavation-induced ground responses of the talus-like rock mass are more predominant by the irregularly-developed backbones formed by polygonal rock blocks, which can be called rock-type talus-like rock mass. When the block content is in a moderate level, i.e., VBP = 50%, the talus-like rock mass exhibits some hybrid characteristics related to both the soil- and rock-type formations, which can be called hybrid-type talus-like rock mass.
(2) The talus-like rock mass behaves differently in the cases of unsupported and supported excavation. For the soil- and hybrid-type talus-like rock masses, the excavation-induced variations in the micro and macro characteristics of an unsupported tunnel are more obvious than those of a supported tunnel. For the rock-type talus-like rock mass, the differences in the two support conditions are inconspicuous. The lining support is essential for maintaining the ground stability of a tunnel excavation in the soil- and hybrid-type talus-like rock masses while the backbones formed by rock blocks in the rock-type talus-like rock mass can provide a certain support for the surrounding ground.
(3) The ratio of cover depth to tunnel diameter (C/d) has a more significant influence on excavation-induced responses of the soil-type talus-like rock mass than the effect of VBP. For the other two types, the VBP plays a dominant role. The microscopic characteristics are more affected by the varying VBP while the excavation-induced ground settlement is more sensitive to C/d. However, there is a strong linkage between the micro and macro characteristics of the excavation-induced ground responses, e.g., the chimney-shaped settlement range agrees well with the void of force chains and reduction ranges of coordination number.

These findings present in our paper have important implications for understanding the ground responses due to the tunnel excavation in this special talus-like rock mass as well as the volumetric block proportion effect on the ground responses, which are beneficial for optimizing the support scheme for underground excavation to achieve a safe and fast construction. In the current model, we used a 2D configuration with a rigid tunnel lining using an assumption of plane strain. In the practical engineering, the ground responses in the longitudinal direction vary over time as the excavation face moves forward and the tunnel lining can deform due to the ground convergence. It is thus recommended to establish a 3D scheme with a flexible tunnel structure to investigate the spatio-temporal responses of the tunnel excavation in the talus-like rock mass in the follow-up studies.

## Figures and Tables

**Table 1:** The calibrated micro parameters for DEM simulations.

Element	Contact Model	Parameter	Value	Unit
Soil particles	Rolling resistance model	Elastic modulus of contacts	70.0	MPa
		Stiffness ratio of normal to tangential contacts	1.5	-
		Friction coefficient	0.5	-
		Rolling resistance coefficient	3.0	-
Rock blocks	Parallel bonding model	Elastic modulus of contacts	100.0	MPa
		Stiffness ratio of normal to tangential contacts	1.5	-
		Friction coefficient	0.45	-
		Elastic modulus of the parallel bond	100.0	MPa
		Stiffness ratio of normal to tangential of the parallel bond	1.5	-
		Parallel bond tensile strength	8.0	MPa
		Parallel bond cohesion	4.0	MPa
		Parallel bond frictional angle	45.0	°
Soil-rock and rock-rock contacts	Rolling resistance model	Elastic modulus of contacts	100.0	MPa
		Stiffness ratio of normal to tangential contacts	1.5	-
		Friction coefficient	0.45	-
		Rolling resistance coefficient	1.0	-

**Table 2:** The numerical modes with different cover depths and tunnel diameters.

Cover Depth (m)	Tunnel Diameter (m)	C/d
10	6.5	1.54
20	7.5	2.67
20	6.5	3.08
20	5.5	3.64
30	6.5	4.62

## Data Availability

Not applicable.
